# NaCl Promotes the Efficient Formation of *Haematococcus pluvialis* Nonmotile Cells under Phosphorus Deficiency

**DOI:** 10.3390/md19060337

**Published:** 2021-06-13

**Authors:** Feng Li, Ning Zhang, Yulei Zhang, Qingsheng Lian, Caiying Qin, Zuyuan Qian, Yanqi Wu, Zhiyuan Yang, Changling Li, Xianghu Huang, Minggang Cai

**Affiliations:** 1 College of Fisheries, Guangdong Ocean University, Zhanjiang 524088, China; lifeng2318@gdou.edu.cn (F.L.); zhangn@gdou.edu.cn (N.Z.); zhangyl@gdou.edu.cn (Y.Z.); lianqingsheng@stu.gdou.edu.cn (Q.L.); tancaiying@stu.gdou.edu.cn (C.Q.); qianzuyuan@stu.gdou.edu.cn (Z.Q.); wuyanqi@stu.gdou.edu.cn (Y.W.); yangzhiyuan@stu.gdou.edu.cn (Z.Y.); licl@gdou.edu.cn (C.L.); huangxh@gdou.edu.cn (X.H.); 2 Fujian Provincial Key Laboratory for Coastal Ecology and Environmental Studies, Xiamen University, Xiamen 361102, China; 3 Key Laboratory of Marine Chemistry and Applied Technology, Xiamen University, Xiamen 361102, China; 4 College of Ocean and Earth Science, Xiamen University, Xiamen 361102, China

**Keywords:** *Haematococcus pluvialis*, astaxanthin, nonmotile cells, phosphorus deficiency

## Abstract

Natural astaxanthin helps reduce the negative effects caused by oxidative stress and other related factors, thereby minimizing oxidative damage. Therefore, it has considerable potential and broad application prospects in human health and animal nutrition. *Haematococcus pluvialis* is considered to be the most promising cell factory for the production of natural astaxanthin. Previous studies have confirmed that nonmotile cells of *H. pluvialis* are more tolerant to high intensity of light than motile cells. Cultivating nonmotile cells as the dominant cell type in the red stage can significantly increase the overall astaxanthin productivity. However, we know very little about how to induce nonmotile cell formation. In this work, we first investigated the effect of phosphorus deficiency on the formation of nonmotile cells of *H. pluvialis*, and then investigated the effect of NaCl on the formation of nonmotile cells under the conditions of phosphorus deficiency. The results showed that, after three days of treatment with 0.1% NaCl under phosphorus deficiency, more than 80% of motile cells had been transformed into nonmotile cells. The work provides the most efficient method for the cultivation of *H. pluvialis* nonmotile cells so far, and it significantly improves the production of *H. pluvialis* astaxanthin.

## 1. Introduction

Astaxanthin is a keto-carotenoid that has a wide range of applications in aquaculture, food, cosmetics, and human health due to its strong antioxidant and coloring functions [1,2]. Currently, there are mainly two types of astaxanthin on the market: artificial synthetic astaxanthin and natural astaxanthin [3]. Artificial synthetic astaxanthin accounts for about 95% of the market and is mainly used in aquaculture [2]. Although synthetic astaxanthin has the advantages of lower cost and price, it has not been approved for human consumption due to possible safety issues [3]. In contrast, natural astaxanthin is significantly better than artificial synthetic astaxanthin in terms of stability, antioxidant activity, absorption effect and biosafety, and has been approved by China, the United States, Japan, and some EU countries for aquaculture, dietary supplements, cosmetic ingredients, and other uses [2,4]. However, due to technical limitations in the production of raw materials, the global production of natural astaxanthin is low, resulting in high market prices [5,6], and the retail prices of nutraceutical grade astaxanthin have even reached US $100,000 per kilogram [7].

*Haematococcus pluvialis* is the most competitive natural source for commercial astaxanthin production and the global annual production capacity is about 800 tons [8], which is still far behind the 10,000-ton for spirulina and 1000-ton for chlorella. The high cell death rate during the production process is the main reason for the low productivity of *H. pluvialis* astaxanthin [9,10]. The accumulation of biomass and the synthesis of astaxanthin are two important factors that must be considered in the production of astaxanthin in *H. pluvialis*. However, culture conditions suitable for rapid cell growth and culture conditions for astaxanthin accumulation are mutually exclusive [4]. A two-stage culture strategy is widely adopted by *H. pluvialis* industry [9], because it is the most effective strategy for solving the contradiction between cell fast growth and astaxanthin accumulation at present [11]. In actual production, however, when the vegetative cells are transferred from the first stage (green stage) to the second stage (red stage), a large number of cell deaths occur due to high intensity of light in combination with nutrient depletion stress. As a result, the overall astaxanthin productivity is very low [9]. Therefore, the reduction of cell mortality in the red stage has become the key to increasing the overall astaxanthin production of *H. pluvialis*.

The cells of *H. pluvialis* usually go through vegetative green stage, intermediate palmella stage and cyst stage, in which several types of cells are distinguished: motile cells, nonmotile palmella cells, and haematocysts (aplanospores) [4]. Previous studies found that the nonmotile cells of *H. pluvialis* were more tolerant to photooxidative stress than motile cells [12]. Using nonmotile cells as the main cell type for the astaxanthin production can significantly reduce the cell mortality and increase astaxanthin productivity in the red stage [13]. However, there are few reports on the induction of *H. pluvialis* nonmotile cells formation. In this study, we first investigated the effect of phosphorus deficiency on the formation of nonmotile cells of *H. pluvialis*, and then the effect of NaCl on the formation of nonmotile cells under the conditions of phosphorus deficiency. Our results showed that the addition of NaCl effectively promoted the formation of nonmotile cells under the condition of phosphorus deficiency. The work provides the most cost-efficient method for the preparation of *H. pluvialis* nonmotile cells so far, and this is of great significance for improving the production of *H. pluvialis* astaxanthin by using cell regulation technology. 

## 2. Results

### 2.1. The Effect of Phosphorus Deficiency on the Formation of Nonmotile Cells of H. pluvialis

**Figure 1:**
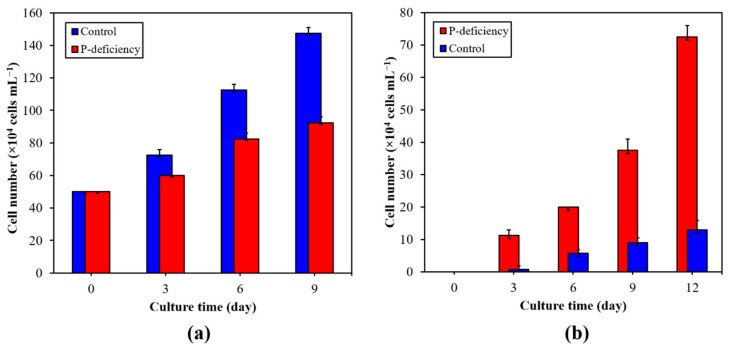
Changes on the total cell number (**a**) and nonmotile cells number (**b**) of *H. pluvialis* in control- and P-deficiency treatment group. The results were presented as mean + SD.

As shown in Figure 1, the total number of cells and the number of nonmotile cells in the two experimental groups both showed a trend of increasing with time. After 9 days of cultivation, the total number of cells and nonmotile cells in the two groups both reached the maximum value. The total number of cells in the control group reached 147.5 × 10^4^ cells mL^−1^, which was about 59% higher than that in the P-deficiency group. The maximum number of nonmotile cells in the P-deficiency group was 37.5 × 10^4^ cells mL^−1^, which was 4.17 times that of the control group. We also calculated the daily percentage growth rate of nonmotile cells, and the results showed that the percentage growth rate of nonmotile cells under phosphorus deficiency condition increased by an average of about 4.5% per day, which was 6.7 times that under normal conditions, indicating that phosphorus deficiency can significantly promote the formation of *H. pluvialis* nonmotile cells.

**Figure 2:**
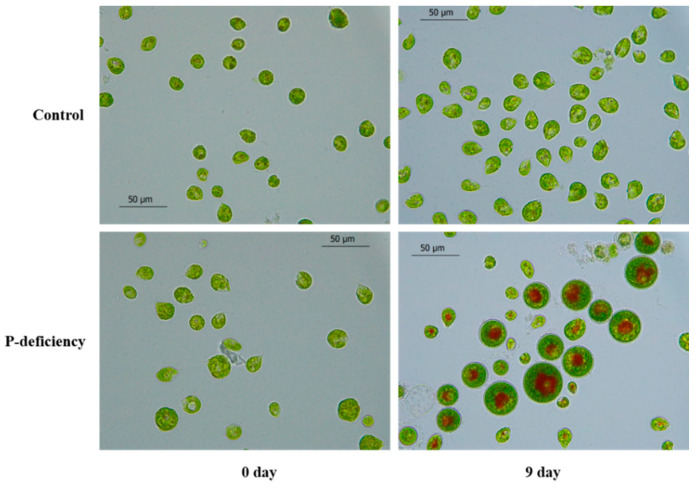
The cell morphology of *H. pluvialis* on day 0 and day 9 in control- and P-deficiency treatment groups.

As shown in Figure 2 and Table 1, after 9 days of phosphorus deficiency treatment, more than 40% of the motile cells have been transformed into nonmotile cells. During this process, red pigmentation due to astaxanthin accumulation appears towards the center of nonmotile cells. By comparison, most of the cells in the control group are still motile cells in green color, with only about 6% nonmotile cells. In addition, some dead cells were observed in P-deficiency group and the cell mortality reached 9.4%, which was about 5.5% higher than that of the control group, indicating that phosphorus deficiency can increase the cell death rate.

### 2.2. The Effect of Adding NaCl on the Formation of Nonmotile Cells under Phosphorus Deficiency Condition

**Figure 3:**
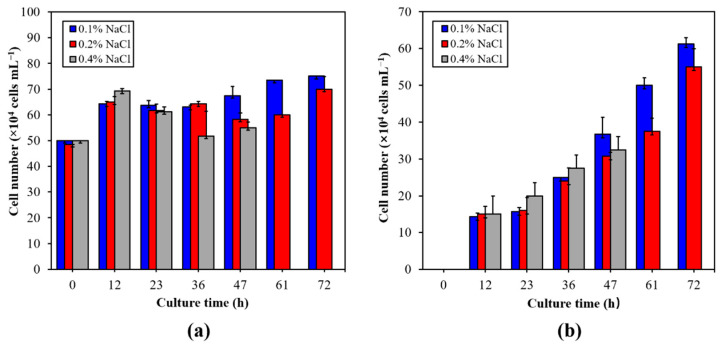
Changes on the total cell number (**a**) and nonmotile cells number (**b**) of *H. pluvialis* in 0.1%, 2%, and 0.4% NaCl treatment groups. The data at 61 h and 72 h in the 0.4% NaCl treatment group are not shown due to cell adhesion has affected the accurate determination of cell number. The results were presented as mean + SD.

Under phosphorus deficiency conditions, adding different concentrations of NaCl has significant effects on the growth, cell morphology, nonmotile cells formation rate, and cell mortality of *H. pluvialis*. As shown in Figure 3a, the total number of cells in both 0.1% NaCl and 0.4% NaCl treatment groups increased first, followed by a decrease, and then increased again. The total number of cells in the 0.2% NaCl treatment group also showed this trend. After 72 h of treatment, the total number of cells in the 0.1% NaCl and 0.2% NaCl treatment groups reached the maximum value, which were 75.0 × 10^4^ cells mL^−1^ and 70.0 × 10^4^ cells mL^−1^, respectively. The maximum total cell number in the 0.4% NaCl treatment group appeared at the 12th h, and the value was 69.25 × 10^4^ cells mL^−1^.

It can be seen from Figure 3b that the number of nonmotile cells in the 0.1% NaCl and 0.2% NaCl treatment groups showed a rapid increase after 23 h of treatment. After 72 h of treatment, the number of nonmotile cells in the 0.1% NaCl treatment group reached 61.25 × 10^4^ cells mL^−1^, which was about 11.4% higher than that in the 0.2% NaCl treatment group. The increase of nonmotile cells number in the 0.4% NaCl treatment group was the fastest among the three groups within 36 h of treatment, and then the increase rate slowed down. After 47 h of treatment, cell adhesion occurred in the 0.4% NaCl treatment group and accompanied by a large number of cell deaths, which made it impossible to accurately determine the cell number. Therefore, the data of the 0.4% NaCl treatment group at 61 h and 72 h were not shown in Figure 3.

**Figure 4:**
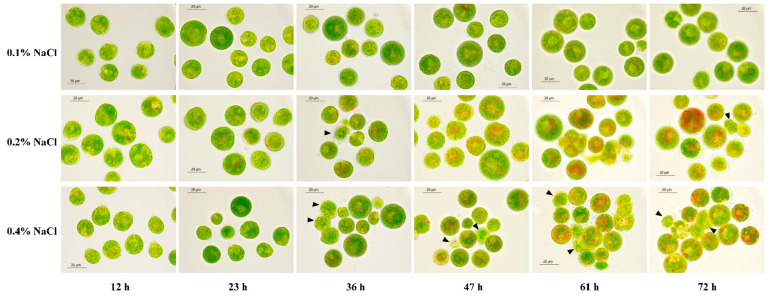
Morphological changes of *H. pluvialis* cells in 0.1%, 0.2%, and 0.4% NaCl treatment groups. The damaged or dead cells are indicated by arrows.

As shown in Figure 4 and Table 2, after 72 h of treatment, 81.7% of the motile cells in the 0.1% NaCl treatment group had been transformed into green nonmotile cells, the daily percentage growth rate of nonmotile cells reached 27.2% day^−1^, and the cells was in good shape. In the 0.2% NaCl treatment group, after 36 h of treatment, the cell color gradually changed from green to orange-green due to the accumulation of carotenoids. After 72 h of treatment, 78.6% of the motile cells transformed into nonmotile cells. The daily percentage growth rate of nonmotile cells was 26.2% day^−1^, and 3.4% of the cells died due to stress.

When the concentration of NaCl added to the phosphorus deficiency medium reached 0.4% (*w*/*v*), it was observed that some cells were damaged after 23 h of treatment. After 36 h of treatment, carotenoids began to accumulate inside the cells. After treatment for 47 h, cell adhesion occurred and over 38% of the cells died (Table 2). By comparison, the cell mortality in 0.1% NaCl and 0.2% NaCl treatment groups were only 1.8% and 3.4%, indicating that high concentrations of NaCl can significantly increase cell mortality rate.

## 3. Materials and Methods

### 3.1. Algal Strain and Culture Conditions

*H. pluvialis* CCMA-451 was obtained from the Center for Collections of Marine Algae in Xiamen University, China. The motile cells were grown photoautotrophically at 20 μmol photons m^−2^ s^−1^ in liquid Bold Basal Medium (BBM) with 3 times NaNO_3_.

The 5-day-old green motile cells were collected by centrifugation (2000 rpm, 2 min), and transferred into a fresh phosphorus-free BBM medium at an initial optical density of 0.5 (OD_680_). Then NaCl was added to the cultures to adjust the concentrations to 1%, 2%, and 4% (*m*/*v*). All experiments were performed in triplicate in a 1-L glass columns (inner diameter 5 cm) at 25 ± 1 °C under continuous illumination (30 μmol photons m^−2^ s^−1^) for 3 days. Culture mixing was provided continuously by bubbling of filtered air enriched with 1.5% (*v*/*v*) CO_2_ at a flow rate of 100 mL min^−1^.

### 3.2. Morphological Observation

The morphological changes of the algal cells were observed using a Leica DM750 light microscope (Leica Microsystems, Wetzlar, Germany) and photos were taken with a Leica ICC50 W camera (Leica Microsystems, Wetzlar, Germany).

### 3.3. Determination of Cell Number

The samples were fixed with Lugol’s iodine solution first. Then, cell numbers were counted using a Neubauer improved cell counting chamber under Leica DM750 light microscope and measured as cells mL^−1^.

The daily percentage growth rate of nonmotile cells (% day^−1^) was calculated as follows:

(1) $$\mathrm{Daily}\;\mathrm{percentage}\;\mathrm{growth}\;\mathrm{rate}\;\mathrm{of}\;\mathrm{nonmotile}\;\mathrm{cells}\;\left({\%\;\mathrm{day}}^{-1}\right)=\frac{\frac{C_{Nt}}{C_{Tt}}\;\times \;100\%}{t}$$

where *C_Nt_* and *C_Tt_* were the nonmotile cells number and total cells number on day t, respectively.

### 3.4. Statistical Analysis

Statistical analysis was performed with the SPSS for Windows statistical software package (IBM SPSS v22.0, Inc., 2010; Chicago, IL, USA). The difference was considered significant when *p* < 0.05 and the results were presented as mean + SD.

## 4. Discussion

The goal of improving the cultivation technology of *H. pluvialis* is to maximize the production of biomass and astaxanthin. Achieving cell growth and astaxanthin accumulation simultaneously under the same cultural conditions is difficult. The major reason for this is that the rapid growth of *H. pluvialis* requires favorable environmental conditions [14,15,16] and the accumulation of astaxanthin requires unfavorable cultural conditions [11,17,18,19]. The two-stage cultivation strategy has effectively solved the contradiction between cell growth and astaxanthin accumulation. However, it fails to solve the high cell mortality during the red stage, so this technology still has a lot of room for improvement.

Previous studies have confirmed that the motile cells are susceptible to photooxidative stress and tend to die under high intensity of light conditions [9,12,20]. Compared with motile cells, nonmotile cells with thick cell walls are not only more tolerant to stress [12], but also more effectively use chemical energy accumulated during cell transformation to rapidly synthesize and accumulate astaxanthin under stress conditions [12,21,22]. Li et al. [20] suggested that using nonmotile cells as the dominant cell type in the red stage can reduce cell mortality more than 70%, while biomass and astaxanthin content can increase up to 2.12 times and 3.5 times, respectively. Therefore, by inducing motile cells to transform into nonmotile cells with more tolerance and astaxanthin accumulation ability before entering the red stage, the problem of high cell mortality is expected to be effectively solved.

The formation of nonmotile cells of *H. pluvialis* is accompanied by encystment, which is considered to be a self defense mechanism [22,23,24]. Previous studies have shown that the encystment of *H. pluvialis* may be related to carbon assimilation [23,25] and phosphorus plays a key role in photosynthetic carbon assimilation [26]. The phosphate translocator is an important structure in the chloroplast membrane, which can transport triose phosphate (the first sugar produced in photosynthesis) produced during the Calvin cycle to the cytoplasm for the synthesis of sucrose, and at the same time transport the released inorganic phosphate (Pi) to chloroplast matrix. When phosphorus is deficient, the Pi exchanged with triose phosphate decreases, resulting in a reduction of Pi in the chloroplast and a decrease in the ATP/ADP ratio, which affects the transport in the C3 pathway and the progress of related reactions, thereby reducing the photosynthetic rate. At the same time, triose phosphate is converted into starch due to phosphorus deficiency and stored in the chloroplast. The reduction of triose phosphate transported from the chloroplast to the cytosol further affects the synthesis of sucrose, thereby inhibiting the photosynthesis of algae and promoting the formation of encystment [14] to maintain the cell integrity, structure, and function. Although phosphorus deficiency can promote the formation of nonmotile cells, the efficiency is low. According to our results, the addition of NaCl under phosphorus deficiency conditions significantly promotes the formation of nonmotile cells. It made the daily percentage growth rate of nonmotile cells increase from 4.5% to more than 26%. We speculate that NaCl promotes the formation of nonmotile cells under the condition of phosphorus deficiency owing to the following reasons. (1) The increase of NaCl concentration affects the oxygen metabolism in algal cells, accelerates the production of reactive oxygen species (ROS), and reduces the function of the scavenging system, which leads to the accumulation of ROS in the cells. (2) Higher salinity increases the osmotic pressure and promotes loss of water from cells, thereby affecting the normal metabolic activities of the cells. (3) The increase of NaCl concentration affects the ion homeostasis in the cells, thereby causing nutritional stress. Thus, it is necessary to carry out further physiological and biochemical studies.

We have to point out that we did not use algae cultured without NaCl for 72 h as a control. However, it doesn’t seem to affect our conclusion that adding NaCl to phosphorus-deficiency medium can significantly promote the formation efficiency of *H. pluvialis* nonmotile cells. We have also determined the most efficient induction method (synergistic induction by phosphorus deficiency and 0.1% NaCl) of nonmotile cells. After three days of cultured under the optimal conditions developed in this study, more than 80% of motile cells transformed into nonmotile cells. The astaxanthin production efficiency of these nonmotile cells should be evaluated by extraction and HPLC in future. This will be profitable for the *Haematococcus* industry.

## Figures and Tables

**Table 1:** The percentage of nonmotile cells, daily percentage growth rate of nonmotile cells, and cell mortality of control- and P-deficiency treatment groups.

Parameters	Control Group	P-Deficiency Treatment
The percentage of nonmotile cells (%)	6.1	40.5
Daily percentage growth rate of nonmotile cells (% day^−1^)	0.67	4.50
Cell mortality (%)	3.9	9.4

**Table 2:** The percentage of nonmotile cells, daily percentage growth rate of nonmotile cells, and cell mortality in 0.1, 0.2, and 0.4 NaCl treatment groups.

Parameters	0.1% NaCl	0.2% NaCl	0.4% NaCl
The percentage of nonmotile cells (%)	81.7 ^1^	78.6 ^1^	59.1 ^2^
Daily percentage growth rate of nonmotile cells (% day^−1^)	27.2	26.2	29.5
Cell mortality (%)	1.8 ^2^	3.4 ^3^	38.2 ^2^

^1^ Obtained after 72 h of treatment. ^2^ Obtained after 47 h of treatment. ^3^ Obtained after 61 h of treatment.
